# Expression of concern: d-Penicillamine functionalized dendritic fibrous nanosilica (DFNS-DPA): synthesise and its application as an innovative advanced nanomaterial towards sensitive quantification of ractopamine

**DOI:** 10.1039/d6ra90028c

**Published:** 2026-03-19

**Authors:** Milad Baghal Behyar, Nasrin Shadjou

**Affiliations:** a Department of Nanotechnology, Faculty of Science and Chemistry, Urmia University Urmia Iran n.shadjou@urmia.ac.ir +98 44 33363311

## Abstract

Expression of concern for ‘d-Penicillamine functionalized dendritic fibrous nanosilica (DFNS-DPA): synthesise and its application as an innovative advanced nanomaterial towards sensitive quantification of ractopamine’ by Milad Baghal Behyar and Nasrin Shadjou, *RSC Adv.*, 2021, **11**, 30206–30214, https://doi.org/10.1039/D1RA05655G.

In the original article, concerns have been raised over the reproducibility and accuracy of the data presented. There are also sections within the manuscript that contradict the results presented in the figures.

In particular, there are concerns over the interpretation of the differential pulse voltammetry (DPV) and chronoamperometry (ChA) results in Fig. S11, as well as the interpretation of the cyclic voltammetry (CV) results in Fig. 2 and Fig. S15.

There are also concerns over the methodology used to obtain the DPV and square wave voltammetry (SWV) results in Fig. S13, as well as the interpretation of those results.

An update will be provided as soon as possible.

Laura Fisher

6th March 2026

Executive Editor, *RSC Advances*

